# Extent, nature and consequences of performing outside scope of training in global health

**DOI:** 10.1186/s12992-019-0506-6

**Published:** 2019-11-01

**Authors:** Ashti Doobay-Persaud, Jessica Evert, Matthew DeCamp, Charlesnika T. Evans, Kathryn H. Jacobsen, Natalie E. Sheneman, Joshua L. Goldstein, Brett D. Nelson

**Affiliations:** 1 Division of Hospital Medicine, Departments of Medicine and Medical Education, Feinberg School of Medicine, Northwestern University, 51 E Huron St, Chicago, IL 60611 USA; 2 Institute for Global Health, Feinberg School of Medicine, Northwestern University, 645 N Michigan Ave, Suite 1058, Chicago, IL 60611 USA; 3 University of California San Francisco, 505 Parnassus Ave, San Francisco, CA 94143 USA; 4 Child Family Health International, 400 29th St, Suite 508, Oakland, CA 94609 USA; 5 Center for Bioethics and Humanities and Division of General Internal Medicine, University of Colorado, 13080 E. 19th Avenue, Aurora, Colorado 80045-2571 USA; 6 Department of Preventive Medicine, Feinberg School of Medicine, Northwestern University, 680 N Lake Shore Dr, Chicago, IL 60611 USA; 7 Department of Global and Community Health, George Mason University, 4400 University Dr, Fairfax, VA 22030 USA; 8 Departments of Neurology, Pediatrics, and Medical Education, Feinberg School of Medicine, Northwestern University, 303 E Chicago Ave, Chicago, IL 60611 USA; 9 Divisions of Global Health and Neonatology, Department of Pediatrics, Massachusetts General Hospital, 125 Nashua St, Boston, MA 02114 USA; 10 Harvard Medical School, 25 Shattuck St, Boston, MA 02115 USA

**Keywords:** Global health, Medical education, Ethics, Scope of training, Scope of practice, Professionalism

## Abstract

**Background:**

Globalization has made it possible for global health professionals and trainees to participate in short-term training and professional experiences in a variety of clinical- and non-clinical activities across borders. Consequently, greater numbers of healthcare professionals and trainees from high-income countries (HICs) are working or volunteering abroad and participating in short-term experiences in low- and middle-income countries (LMICs). How effective these activities are in advancing global health and in addressing the crisis of human resources for health remains controversial. What is known, however, is that during these short-term experiences in global health (STEGH), health professionals and those in training often face substantive ethical challenges. A common dilemma described is that of acting outside of one’s scope of training. However, the frequency, nature, circumstances, and consequences of performing outside scope of training (POST) have not been well-explored or quantified.

**Methods:**

The authors conducted an online survey of HIC health professionals and trainees working or volunteering in LMICs about their experiences with POST, within the last 5 years.

**Results:**

A total of 223 survey responses were included in the final analysis. Half (49%) of respondents reported having been asked to perform outside their scope of training; of these, 61% reported POST. Trainees were nearly twice as likely as licensed professionals to report POST. Common reasons cited for POST were a mismatch of skills with host expectations, suboptimal supervision at host sites, inadequate preparation to decline POST, a perceived lack of alternative options and emergency situations. Many of the respondents who reported POST expressed moral distress that persisted over time.

**Conclusions:**

Given that POST is ethically problematic and legally impermissible, the high rates of being asked, and deciding to do so, were notable. Based on these findings, the authors suggest that additional efforts are needed to reduce the incidence of POST during STEGH, including pre-departure training to navigate dilemmas concerning POST, clear communication regarding expectations, and greater attention to the moral distress experienced by those contending with POST.

## Background

Alongside the increasing globalization of the world’s modern economies, cultures, and populations there has been a parallel globalization in healthcare, with countless clinicians and organizations working across international borders. Indeed, every year, thousands of healthcare professionals and medical trainees engage in short-term experiences in global health (STEGH) [1–7]. Ranging in duration from 1 week to a few months, STEGH typically involve health professionals from high-income countries (HICs) traveling to low- and middle-income countries (LMICs) for educational, training, capacity-building, or research purposes.

Ethical and best practice guidelines emphasize that such programs should primarily and sustainably benefit the local community [3, 7–12]. This emphasis arose out of the recognition that STEGH can burden communities, fail to meet expectations, and even cause unintentional harms [3, 7, 9, 12–20]. One of the many ethical challenges associated with STEGH arises when visiting clinicians and trainees are faced with opportunities or requests to perform outside the scope of their training [3, 8, 21–28]. Locally licensed clinicians working in LMICs often have a broader scope of practice than clinicians who work in HICs, where specialty practice is common. Existing literature around STEGH provides qualitative and case-based evidence that performing outside scope of training (POST) can be a particular challenge for HIC professionals and trainees operating in unfamiliar clinical and cultural environments and working under resource constraints [17, 21, 23, 27, 29–31]. In addition, there is growing evidence that pre-health students seek out the opportunity to practice beyond their scope of training due to curiosity, eagerness for clinical exposure, and belief that it will improve their likelihood of gaining admission to their desired schools. Despite ethical guidelines recommending against POST, exigent circumstances such as humanitarian emergencies, critical personnel shortages, or the absence of referral options may seem to require some level of POST. Even then, POST requires careful consideration of context, competence, comfort, and circumstances [3, 7, 8, 22, 26, 32–34]. In practice, POST can reveal the tension between the duty to provide care and the imperative to do no harm [8, 26, 32, 35]. Frameworks to aid in ethical decision-making around scope of practice have been proposed, but prior research has focused on qualitative case studies of trainee groups or professionals working in humanitarian settings [3, 5, 7, 9, 16, 24, 28, 32, 35–38]. Whether existing frameworks apply to health professionals more broadly or to less acute situations encountered during STEGH remains unknown.

The goals of this study were to clarify the circumstances under which health professionals and trainees face requests to perform outside their training during STEGH, and to explore the perceived impact of POST on practitioners.

## Methods

### Survey design

A research team composed of experienced clinical and global health faculty designed a survey instrument including 39 closed- and open-response questions about demographics, STEGH during the past 5 years, and POST (Additional file 1). STEGH locations were classified based on the World Health Organization definitions for health system levels [39]. Global health faculty and trainees at Northwestern University Feinberg School of Medicine, Harvard Medical School, and the University of California, San Francisco School of Medicine pilot-tested the survey for content validity. The draft questionnaire was revised based on their feedback. The survey instrument and an abridged report of limited findings have been previously described elsewhere [40].

The study protocol was approved by the institutional review board of Northwestern University (Protocol STU00205018).

### Recruitment and data collection

Data were collected online between April and July 2017. Survey participants were recruited via convenience sampling at global health-related academic conferences, within professional networks, and via email listservs, including the Consortium of Universities for Global Health, American Academy of Pediatrics, American Academy of Family Practitioners, Global Emergency Medicine Academy, and Society for Hospital Medicine. Recipients could forward the link to the online survey, resulting in additional participants recruited through snowball sampling. Responses were collected via Qualtrics (Provo, UT, USA). Respondents were allowed to proceed with the survey if they self-identified as being at least 18 years of age and having participated in STEGH in an LMIC or another resource-constrained setting within the preceding 5 years.

### Quantitative data analysis

Univariate statistics including means, medians, frequencies, percentages, and standard deviations were used to describe quantitative survey responses. A variable for “returner status” was developed, grouping “non-returners” who never returned to the same STEGH site, “returners” who went to the same STEGH site 2–5 times, and “frequent returners” who went to the same STEGH site six or more times in the preceding 5 years. Bivariate analyses using chi-square statistics were used to examine associations between demographic and POST variables. All quantitative analyses were conducted using SAS, version 9.4 (SAS Institute, Cary, NC, USA).

### Qualitative data analysis

An emergent thematic content analysis approach was used to code qualitative responses [37]. First, two researchers (JE, MD) reviewed more than half of the open-ended responses line-by-line and developed a set of preliminary codes. Because many of the responses included emotional overtones, an emotive style of coding was pursued [41]. Related sections of text were grouped into categories based on the preliminary codes and emergent patterns in the data. After two iterations of this process, a codebook was developed. Next, coding was undertaken independently by two other researchers (ADP, KHJ) who reviewed the free text responses line-by-line and assigned codes from the final codebook. To help ensure inter-coder reliability, the two researchers discussed and resolved coding discrepancies. Coding disagreements were resolved by a third researcher (JE) through negotiated consensus. To ensure accuracy, the original coders then reviewed and confirmed the assigned codes for half of the line-by-line transcripts. Reflexivity techniques, such as being open and transparent about the researchers’ own views, were used through the coding process. The coders’ own perspectives as individuals with expertise in global health education and/or ethics related to STEGH, and hence familiarity with existing guidelines, are important to acknowledge.

## Results

**Fig. 1:**
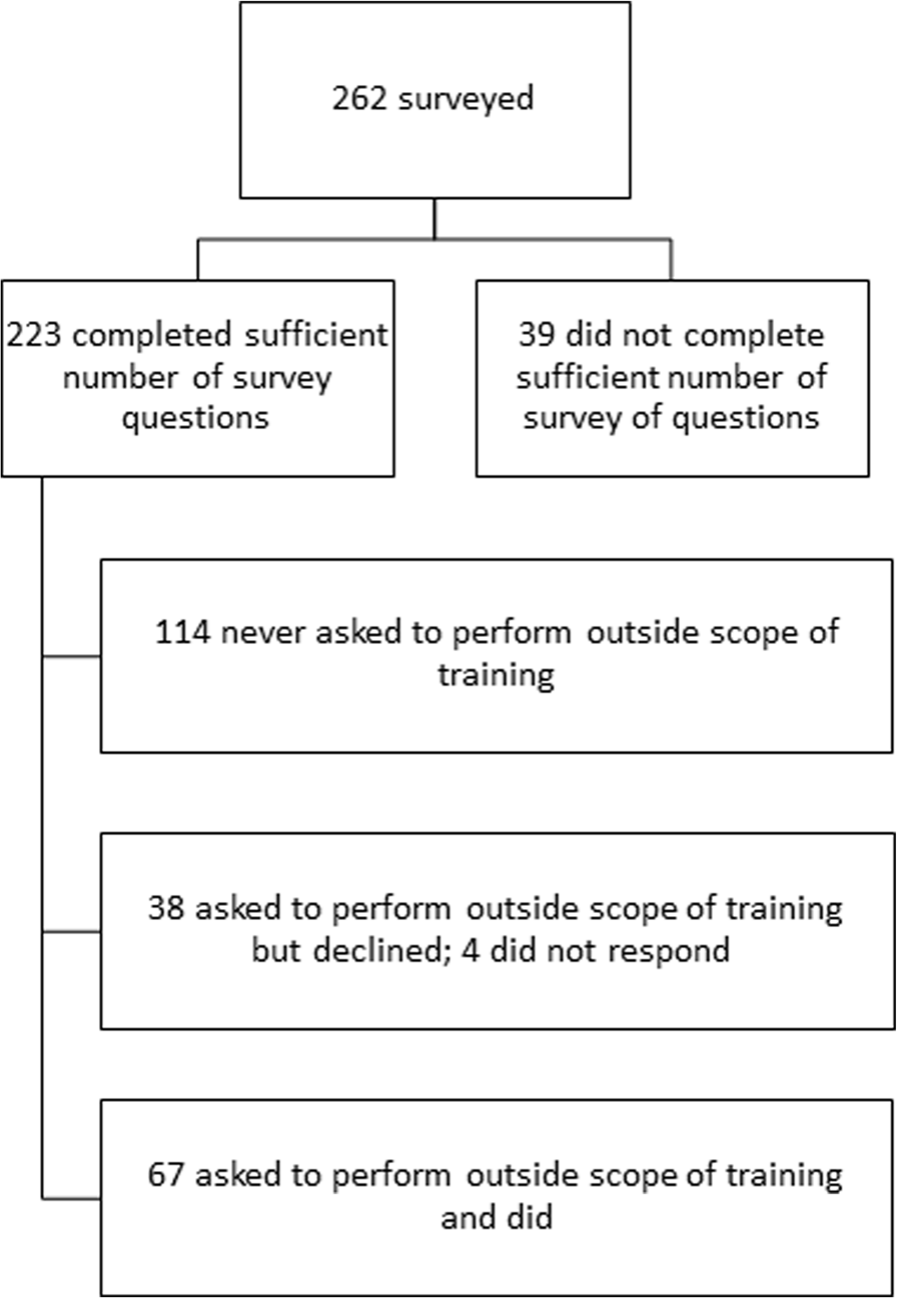
Study Participant Inclusion Chart

A total of 262 survey responses were received. The final analysis included the 223 respondents who answered at least one question about POST (Fig. 1). Respondents spent a median of 28 days participating in STEGH in the preceding year and 30 days annually during the preceding 5 years. Many respondents had spent several months on STEGH, so the mean (standard deviation) was much higher: 73 days (105) in the past year and 80 days (102) annually over the past 5 years. More than 80% of the respondents had made several visits to the same site for STEGH. The typical respondent was in the medical field (82%), had trained in North America (84%), was a licensed health professional (66%), and had an intentional clinical focus (86%) during STEGH in LMIC settings (Table 1).

**Table 1:** Survey Respondent Demographics (*N* = 223)

Demographic Description		*n*	%
Degree^a^	MD/DO	183	82.1
	RN	8	3.6
	Physician assistant/nurse practitioner	8	3.6
	Other	24	10.8
Medical Discipline^a^	Internal or Family Medicine	62	27.8
	Other specialties	161	72.2
Professional Status^a^	Licensed practitioner	146	65.5
	Trainee	65	29.1
	Fellow	15	6.7
	Resident	34	15.2
	Medical student	15	6.7
	Other clinical field	1	0.4
	Other	12	5.4
Training Region	North America	134	84.3
	Other	25	15.7
	Latin American/Caribbean	7	4.4
	South Asia	5	3.1
	Europe and Central Asia	4	2.5
	Sub-Saharan Africa	4	2.5
	Middle East and North Africa	1	0.6
	Unknown	64	–
STEGH Professional Role^a^	Clinical	191	85.7
	Non-clinical	32	14.4
STEGH World Bank Region^a^	Sub-Saharan Africa	109	48.9
	Latin American and Caribbean	107	48.0
	South Asia	44	19.7
	East Asia and Pacific	25	11.2
	North America	17	7.5
	Middle East and North Africa	12	5.4
	Europe and Central Asia	7	3.1
STEGH Location^a^	Urban	135	60.5
	Rural	115	51.6
	District/village	91	40.8
	Peri-urban	41	18.4
	Other	4	1.8
STEGH Organization Type^a^	NGOs and other non-profits	143	64.1
	University	99	44.4
	Governmental	58	26.0
	International bilateral, multilateral, and health care foundations	28	12.6
	Other	13	5.8
STEGH Care Setting^a^	Specialty hospital or national referral center	94	42.2
	Primary level or district hospital	93	41.7
	Community health workers in home or village setting	73	32.7
	Primary care clinic	68	30.5
	Health center or dispensary	37	16.6
	Other	23	10.3
Returned to STEGH location	Non-returner (1 trip)	19	8.5
	Returner (2–5 trips)	116	52.0
	Frequent returner (6 or more trips)	63	28.3
	No response	25	11.2

^a^Categories were not mutually exclusive

### Requests to perform outside scope of training

Respondents perceived that requests to perform outside their scope of training occurred often during STEGH. Nearly half (49%) of survey respondents reported that they were asked to perform beyond their scope during STEGH in the preceding five years (Table 2). Of these, 37% were asked 1–3 times, 30% were asked 4–10 times, and 33% more than 10 times. Nearly every demographic group that participated in the study reported a high rate of being asked to perform outside scope of training (Table 3), but respondents identified some variation by setting. For example:

“[It] largely depends on the setting. A large academic center in an LMIC with multiple international collaborations, for example, may be better equipped to manage and assign duties to rotating medical students, residents, and faculty without causing an ethical dilemma. However, a site that is severely understaffed with few international connections may ask more of visitors… Out of the various experiences I have had abroad, [being asked to perform outside scope of training] was most apparent at a small, rural clinic in [Country A]. During my time at a large hospital in [Country B], however, I rarely had the problem arise.” (Allopathic medical student)

**Table 2:** Perceptions, Prevalence, and Reasons Related to POST by Health Professionals During STEGH

Survey Question	Response	n/N	%
How often do HIC professionals perform clinically beyond their scope of training?	Always	4/223	1.8
	Frequently	81/223	36.3
	Sometimes	75/223	33.6
	Infrequently	48/223	21.5
	Never	15/223	6.7
Do you believe it is appropriate for HIC-trained clinicians to perform beyond their scope of training?	Yes	13/223	5.8
	No	65/223	29.2
	It depends	123/223	55.2
	No response	22/223	9.5
Were you ever *asked* to perform clinical activities beyond your scope of training?	Yes	109/223	48.9
*If yes, number of times:*	1–3	39/105	37.1
	4–10	31/105	29.5
	> 10	35/105	33.3
	No response	4/109	3.7
Did you ever *perform* clinical activities beyond your scope of training?	Yes	67/109	61.4
*If yes, number of times:*	1–3	27/64	42.2
	4–10	19/64	29.7
	> 10	18/64	28.1
	No response	3/67	4.5
Why do you feel you were in a situation or situations in which you practiced clinically beyond your scope of training? (May select more than one answer.)			
My training did not match my host’s expectations		25/67	37.3
I had an inadequate level of supervision in-country		14/67	20.9
I was inadequately prepared to decline practicing beyond my scope of training		13/67	19.4
I perceived an inadequate level of available staff, equipment, or resources		9/67	13.4
I overestimated my own capabilities		5/67	7.5
I wanted to be able to perform a procedure/technique I was not very familiar with		5/67	7.5
I did not seek adequate assistance when I needed it		1/67	1.5
Other		13/67	19.4

**Table 3:** Bivariate Analysis of Survey Responses About POST During STEGH

Category		Asked to Perform Outside Scope of Training % (n/N)	Did Perform Outside Scope of Training % (n/N)	Believes it is or could be appropriate to Perform Outside Scope of Training% (n/N)
Total		48.9 (109/223)	54.3 (57/105)	61.0 (136/223)
Degree	MD/DO	48.1 (88/183)	60.0 (51/85)	62.8 (115/183)
	Others	52.5 (21/40)	80.0 (16/20)	52.5 (21/40)
Medical Discipline	Internal or Family Medicine	45.2 (28/62)	59.2 (16/27)	62.9 (39/62)
	Other Specialties	50.3 (81/161)	65.4 (51/78)	60.3 (97/161)
Professional Status	Licensed practitioner (MD/DO)	44.7 (59/132)	50.9 (29/57)	68.9 (91/132)
	Resident/Fellow	55.1 (27/49)	80.8 (21/26)	49.0 (24/49)
	Medical student	66.7 (10/15)	100.0 (10/10)	53.3 (8/15)
	Other	48.2 (13/27)	58.3 (7/12)	48.2 (13/27)
STEGH Professional Role	Clinical	52.9 (101/191)	63.9 (62/97)	64.9 (124/191)
	Non-clinical	25.0 (8/32)	62.5 (5/8)	37.5 (12/32)
STEGH Location	Urban	42.2 (57/135)	59.7 (34/57)	65.2 (88/135)
	Rural	45.2 (52/115)	65.4 (34/52)	73.0 (84/115)
	District/Village	46.2 (42/91)	59.5 (25/42)	67.0 (61/91)
	Periurban	43.9 (18/41)	61.1 (11/18)	53.7 (22/41)
STEGH Organization Type	NGO or non-profit	45.5 (65/143)	63.1 (41/65)	69.9 (100/143)
	University	39.4 (39/99)	64.1 (25/39)	59.6 (59/99)
	Government	55.2 (32/58)	59.4 (19/32)	62.1 (36/58)
	International bilateral	33.3 (4/12)	50.0 (2/4)	66.7 (8/12)
	Foundation	27.3 (3/11)	66.7 (2/3)	90.9 (10/11)
	Multilateral	0.0 (0/5)	0.0 (0/0)	100.0 (5/5)
STEGH Care Setting	Specialty hospital or national referral center	45.5 (43/94)	65.1 (28/43)	63.8 (60/94)
	Primary level or district hospital	47.3 (44/93)	70.5 (31/44)	65.6 (61/93)
	Community health care setting	35.6 (26/73)	53.9 (14/26)	71.2 (52/73)
	Primary care clinic	50.0 (34/68)	67.7 (23/34)	76.5 (52/68)
	Health center or dispensary	43.2 (16/37)	56.3 (9/16)	67.6 (25/37)
Returned to STEGH Location	Non-returner (1 trip)	52.6 (10/19)	60.0 (6/10)	73.7 (14/19)
	Returner (2–5 trips)	40.5 (47/116)	53.2 (25/47)	65.5 (76/116)
	Frequent-returner (6+ trips)	49.2 (31/63)	74.2 (23/31)	69.8 (44/63)

### Engaging in POST

Of those asked to perform outside their training, 61% (67/109)—30% of all respondents—reported engaging in POST. Forty-two percent of this subset indicated doing so 1–3 times over the prior 5 years, 30% reported 4–10 times, and 28% more than 10 times (Table 2). Medical students (100%) and residents and fellows (81%) reported higher rates of POST than licensed physicians (51%) (*p* = 0.001) (Table 3). The most frequently performed POST procedures included basic ultrasound (15%), fracture management (12%), wound care and suturing of lacerations (12%), endotracheal intubation (9%), vaginal delivery (9%), and neonatal resuscitation (9%). Of the 67 respondents who did engage in POST, 33% indicated that it was “very” or “completely” likely that they would make the same decision in similar situations; however, only 15% felt “very” prepared to manage requests to perform outside their scope..

### Perceptions about appropriateness of and reasons for POST

Sixty percent (136/223) of respondents reported that it is or could be appropriate for HIC health professionals participating in STEGH to perform beyond their scope of training in some situations (Table 2). Licensed practitioners (69%) were more likely than others (49%) to believe that POST is or could be appropriate (*p* = 0**.**004) (Table 3). When offered a list of potential factors that might contribute to POST, respondents identified several common factors, including mismatch between hosts’ expectations and visiting health professionals’ training (37%), a suboptimal level of supervision at the host site (21%), inadequate preparation to decline POST (19%), and perception of an inadequate level of available staff, equipment, or resources (13%) (Table 2).

Those who believed POST was appropriate often cited emergency situations and a perceived lack of alternative options.

“When there are no alternative providers and the situation clinically requires it, it may be the only option available to a patient.” (Licensed MD, Internal Medicine).

“For elective cases without imminent danger, clinicians should not practice beyond their scope. However, in emergencies, there may be no other alternative. This constitutes a more challenging ethical scenario.” (Resident MD, General Surgery).

“Sometimes there are situations that cannot be controlled. If someone is near death on the side of a road, for example, we would not blame a layperson for attempting to help.” (Allopathic medical student).

A minority of survey respondents commented that each situation required a consideration of risks and benefits, as well as local capacity.

“Risks and benefits of providing care beyond the scope of training needs to be well accounted for. If there are other providers who are appropriately trained to provide that care, then HIC-trained clinicians should not go beyond their level of training to offer a certain care. However, if the alternative to offering this care is a high risk of an adverse health event, then I believe it is appropriate for any clinician to do their best in caring for patients as needed.” (Resident MD, Internal Medicine).

Some respondents provided reflections about the challenges their peers encounter when managing care in unfamiliar settings, observing that visiting clinicians often have a limited understanding of the cultural context, the health system, and available resources.

“HIC-trained clinicians often underestimate the capacity and availability of physicians in LMIC settings. They also often fail to completely understand the cultural and structural aspects of the medical systems within these settings.” (Resident MD, Obstetrics/Gynecology).

“[Visiting clinicians] need to consider urgency of situation and alternatives. Often the person from the HIC is not in the best position to judge this without understanding environment, culture, language. So such decisions should not be taken lightly.” (Licensed MD, Family Medicine).

“For trainees, it is where to draw the line with what constitutes adequate supervision when allowing the resident to operate. For professionals in general it is when to say ‘no’ to an operation that may be outside of a surgeon’s comfort level or one that is made riskier by the constraints of the environment.” (Resident MD, General Surgery).

Respondents also provided details about factors that may contribute to being asked to perform or deciding to perform outside of scope.

“Local understanding of limited scope—the sense that just because I’m a doctor doesn’t mean that I can fix everything or see every kind of patient.” (Licensed MD, Pediatrics).

“[When working abroad,] I sometimes performed beyond my scope of training in the U.S. but in accordance with what my scope of training would be expected to be in the country I was in.” (Resident MD, Pediatrics).

### Reactions to and sentiments about POST

Qualitative responses described a wide range of emotional responses related to POST, including anxiety, anger, frustration, and excitement. The majority of respondents who reported engaging in POST expressed negative emotions as a result (Table 4). When asked to describe their current feelings about past experiences, some who had engaged in POST cited persistent negative emotions, such as “guilt, frustration, remorse, discomfort, fear, and stress.” Others reported acceptance of the outcome, describing POST as “the best thing that was possible” or “the right thing in the moment.”

**Table 4:** Selected Examples of Health Professionals’ Emotional Reactions and Sentiments Related to POST during STEGH

Emotion	Illustrative Quote	Respondent
Anxiety	“I was anxious. There seemed to be no easy answer of what was the best thing to do.”	MD Fellow, Internal Medicine and Pediatrics
Frustration	“I felt overwhelmed by the responsibility, terrified I was going to give suboptimal care that could result in death, and angry/frustrated that I was in the position of providing care beyond my scope or not providing care to these infants.”	Licensed advanced practice provider, Obstetrics/Gynecology
Discomfort	“It is uncomfortable. You have years of training and are often looked to as the expert, but in reality, you have not been training in such activities and do not have the skills to complete such tasks. You don’t want to stand by and do nothing, but at the same time you don’t want to do more harm than good. I am often left feeling incredibly inadequate and inept.”	MD Fellow, Pediatrics
Remorse	“If I didn’t do something, the patient would have a worse outcome. Something was better than nothing… I don’t regret it, but I wish it ended differently, since the patient died.”	MD student
Excitement	“Excited… Everybody should be exposed to such challenges especially in LMICs.”	Licensed MD, Plastic Surgery
Conflicted	“Torn. On one hand, not appropriate. On the other hand, if I didn’t do it, who would?... [I felt] bad. It was unfair to the patients.”	MD Fellow, Obstetrics/Gynecology

When asked to describe the most challenging situation in which respondents engaged in POST, responses reflected the theme of “a lack of alternatives,” including perceived personnel shortage, lack of resources, and clinical urgency.

“[I was] asked to perform an arthrocentesis on a patient when this was not a skill I had previously performed. I did so due to a lack of alternate providers.” (Licensed MD, Internal Medicine).

“Working outside your usual scope of practice [occurs] because there are limited specialists or referral options.” (Licensed MD, General Surgery).

“I had to attempt to perform a pars plana vitrectomy as a last-ditch effort to save the eye, as our retina surgeon was back in the U.S. on furlough and the patient wasn’t willing to go to the capital city four hours away... I was not successful in saving the vision. While I think the bad outcome of this was set regardless of whether I did something, did not do something, or whether I sent the patient to the capital city or not, I felt very out of my comfort zone.” (Licensed MD, Ophthalmology).

Some clinicians described being asked to use knowledge or skills in which they had partial training at some point in time, typically in medical school, but that were not part of their current expertise.

“Sometimes I have to reach back to medical school training and improvisation to get some things done. I see and do a greater variety of clinical scenarios when overseas but still feel like I am relying on past training when I commit to treatment/intervention. Occasionally I have felt pressured to make clinical decisions that I felt underprepared to make—usually due to the combination of the patient scenario and lack of info/diagnostics that would be helpful.” (Licensed MD, Pediatrics).

“As an internal medicine resident, I saw and cared for children... Both myself and the attending did not have training apart from medical school in pediatrics.” (Resident MD, Internal Medicine).

Some respondents suggested that students participating in STEGH expect to perform outside their scope of training, and that opportunities to engage in POST are part of the strategy for recruiting volunteers and sustaining programs.

“[A challenge is] providing a meaningful experience for the students that will encourage them to speak positively about the program (to ensure continuation of the program) without subjecting the populations in the LMIC setting to undue harm. Often there is less concern in the general population of these areas for clear consent and patient self-advocacy, so pre-med students are often presented as higher ranking or more knowledgeable than they actually are.” (Allopathic medical student).

The opportunity costs of POST were also noted, and some respondents postulated that efforts to address immediate clinical situations may compromise capacity-building and health systems strengthening.

“Too often professionals or trainees feel obligated to provide clinical care based on their own standards and cultural norms that are either out of proportion or not aligned with local customs and practices, misuse local resources, or [are] out of the realm of their scope of practice to feel good about themselves helping patients who are in need of care. Instead, their efforts, energy, and resources could be directed at improving the clinical skills and capacity of local infrastructure to provide the same level of care they wished was being done so many more could be helped within the confines of the local health system, according to cultural practices and prioritize the local resources appropriately.” (MD Fellow, Pediatrics).

## Discussion

Situations in which visitors may feel pressure or justification to perform beyond their scope have been identified as one of the major ethical dilemmas associated with STEGH [12, 16, 23–27, 29, 30, 33, 37, 42–44]. POST is generally contradictory to professional guidelines, risks patient safety, and may violate national laws and regulations; understanding this phenomenon is of critical importance. Although POST has been described as a major ethical challenge in international health practice, previous research about POST in the context of STEGH is limited [3, 5, 7, 9, 16, 24, 28, 32, 35–38]. Despite calls to action for more intensive pre-departure ethics training and stronger partnerships for STEGH [13, 28, 32, 45, 46], few studies have actually examined POST among the large population of health professionals and trainees participating in STEGH.

This study is the first to systematically characterize the prevalence of POST during STEGH and to elucidate the circumstances and emotional ramifications of POST. As such, our study offers important insights for interventions that might reduce inappropriate practices and, by extension, reduce unintended harms.

This study provides compelling new evidence that HIC health professionals and trainees participating in professional activities during STEGH are asked to and do perform outside their scope of training with high frequency. Nearly half of study participants reported being asked to perform beyond their scope and more than 60% of those respondents engaged in POST. Our results also highlight that POST is not merely an issue for clinicians, as one in four respondents involved in non-clinical activities during their STEGH were asked to perform clinical activities outside their training. POST is a challenge for everyone, no matter what type of host organization or practice site they select [3, 7, 8, 47]. Findings from our study make apparent a need for expanded access to and uptake of high-quality education about POST, as well as professional guidelines for responding to requests, across the breadth of STEGH activities and participants.

Understanding why POST occurs is critical to designing interventions that will reduce its occurrence. Our respondents felt that POST was a common practice during STEGH, and its commonality may foster a sense of its normalcy. Respondents also identified a mismatch between hosts’ expectations and visiting health professionals’ training, suboptimal supervision, and inadequate preparation to decline POST as among the most important situational factors leading to POST. While emergencies and resource shortages cannot always be avoided, mismatched expectations can be addressed, supervision arranged, and management of ethical and moral challenges prepared for in advance. These may be more effective strategies for mitigating POST than attempting to modify beliefs about its appropriateness. Indeed, in our study population, such beliefs were not strongly associated with decisions about POST, one way or the other.

Finally, the emotionally and morally distressing dimensions of POST cannot be overlooked. The persistence of negative feelings about past experiences with POST underscores the need not only to prepare individuals to handle requests, but to debrief about those situations afterwards, with special consideration given to those who have been exposed to highly traumatic events.

This study has several limitations. First, North Americans were disproportionately represented, so the results primarily reflect the attitudes and experiences of individuals from North America. Second, by necessity our survey was developed de novo and did not include validated items. Third, the use of convenience and snowball sampling prevented a determination of the full number of potential participants who were contacted and the calculation of a response rate. Fourth, there is the possibility of response bias, skewing our sample towards those with experience involving POST. Fifth, for both the survey and open-ended items, it is possible that recall bias (e.g., respondents’ remembering negative experiences over positive ones) and social desirability bias (e.g., respondents’ reporting negative emotional experiences over positive ones because they believe that is what researchers want to hear) influenced our findings. Lastly, despite our efforts at minimizing the effect of the researchers’ own subjective biases in the qualitative analysis through reflexivity, as for all qualitative studies, this subjectivity could have affected our findings.

In summary, our study participants have, cumulatively, decades of experience working in health settings with limited resources and partnering with international host organizations for STEGH. And yet, despite this extensive experience, one of the most basic ethical issues encountered during STEGH continues to elude straightforward solutions. Researchers in global health ethics have proposed several frameworks for navigating dilemmas that might arise during STEGH, including requests for POST [3, 7, 9, 24, 32, 34–36, 38, 48]. We agree with existing recommendations that pre-departure training should be required; that expectations, scope of training, and necessary supervision should be established prior to starting STEGH; that activities should be undertaken with a commitment to local capacity-building; and that the burden of educating visitors about the local clinical, legal, and cultural environment should not fall disproportionately on the hosts. However, improved recommendations and frameworks that specifically address POST are still needed [26, 32, 35, 49]. We also recognize that while we can create better models to aid in decision-making, the outcomes of ethically challenging situations can never be absolutely certain. The goal should be to close the gap between providing necessary care and doing unintentional harm.

## Conclusions

Our study is one of the first to delve into the context and consequences of POST during STEGH, but it should not be the last. There is an urgent need for additional research on POST that explores these circumstances in greater detail, so that students, professionals, organizations, and partnerships are able to make informed decisions about creating, funding, and participating in these programs. We should prioritize the highest principles and aims of global health, emphasizing sustainability and safety. It is time for an honest reckoning with the challenges of POST.

## Supplementary information

**Additional file 1.** Qualtrics survey instrument.

## Data Availability

The datasets used and analyzed during the current study are available from the corresponding author on reasonable request.

## Abbreviations

HIC: High-income countries
LMIC: Low- and middle-income countries
POST: Performing outside one’s scope of training
STEGH: Short-term experiences in global health
