# Supplementary material for: Extent, nature and consequences of performing outside scope of training in global health
Source: Global Health. 2019 Nov 1;15:60. doi: 10.1186/s12992-019-0506-6 (PMC6823963; doi:10.1186/s12992-019-0506-6)
Supplement: Supplementary file 1 — Additional file 1. Qualtrics survey instrument. [file 12992_2019_506_MOESM1_ESM.pdf]

Skills and Scope: Ethical Practice of Clinical Medicine in LMIC and Resource-Constrained Settings

Consent

WELCOME LETTER AND CONSENT:

The purpose of this research is to understand health professional activities with regard to scope of training when practicing in low and/or middle-income country (LMIC) or resource-constrained settings.

You are invited to participate in this research project because you are likely to have undertaken professional activities in LMIC or resource-constrained settings outside of your country of training. Your participation in this research study is completely voluntary. You can discontinue your participation at any time without being penalized.

As part of this research you will be asked to complete an online survey that will take approximately 15 minutes. In the survey you will be asked questions about your professional activities in LMIC or resource-constrained settings, your opinions about the clinical skills required to practice in such settings, the types of procedures you have performed or have been asked to perform while working in these settings, and your responses to scenarios in which you were asked to practice beyond your scope of training.

To help protect your confidentiality, the surveys will not record information that will personally identify you, such as your name or IP address. However, if you agree to be contacted at the end of the survey, your e-mail address will be recorded in a secure database.

The results of this study will be used to further understanding about scope of training and professional activities by those trained outside of LMICs while in LMICs.

Thank you for your time,
Ashti Doobay-Persaud, MD, Northwestern University Brett Nelson, MD, MPH, DTM&H, Harvard University Jessica Evert, MD, Child Family Health International, University of California, San Francisco Laurel Gabler, MD, MPH, Children's Hospital of Philadelphia Phuoc Le, MD, MPH, University of California, San Francisco Natalie Sheneman, Northwestern University
Joshua Goldstein, MD, Northwestern University **If you have any questions about the research study, please contact:**
Ashti Doobay-Persaud, MD
645 N Michigan Ave Ste 1058
Chicago, IL 60611
a-doobay-persaud@northwestern.edu
312-503-8816

ELECTRONIC CONSENT: Please select your choice below.

Clicking on the "agree" button below indicates that:
- **You have read the above information**
- **You voluntarily agree to participate**
- **You are at least 18 years of age**

If you do not wish to participate in the research study, please decline participation by clicking on the

"disagree" button.

- AGREE: I agree to participate in this study
- DISAGREE: I do not want to participate in this study

Definitions

For the purposes of this survey, please use the following definitions:

- **Resource-Constrained Setting: Settings that are subject to limitations on equipment, personnel, local infrastructure, and other resources that affect health, health care, and health care delivery.**
- **Low and/or Middle-Income Country (LMIC): For the current 2017 fiscal year, low-income economies are defined as those with a gross national income (GNI) per capita, calculated using the World Bank Atlas method, of $1,025 or less in 2015; lower middle-income economies are those with a gross national income per capita between $1,026 and $4,035.***
- **High-Income Country (HIC): For the current 2017 fiscal year, high-income economies are those with a GNI per capita, calculated using the World Bank Atlas method, of $12,476 or more.***

***World Bank. (2017). World Bank Country and Lending Groups. Retrieved from https://datahelpdesk.worldbank.org/knowledgebase/articles/906519-world-bank-country-and-lending-groups**

Have you performed health professional activities or other professional activities in a LMIC or resource-constrained setting within the last 5 years?

- Yes
- No

Degree/Title

Please indicate your degree(s).

- Doctor of Medicine
- Doctor of Osteopathic Medicine
- Advanced Practice Provider (Physician Assistants and Nurse Practitioners)
- Registered Nurse
- Not applicable/still in training
- Other

Which of these best describes your current professional status. Please select one option.

- Resident
- Fellow
- Licensed practitioner (completed training)

- Medical student
- Other health professional student
- Other

In what country did you complete your health professional training?

In what country are you completing your health professional training?

What is your specialty? Select all that apply by holding down the CTRL button (on a PC) or the Command button (on a Mac) while making your selection.

- Allergy/Immunology
- Anesthesiology
- Dermatology
- Diagnostic Radiology
- Emergency Medicine
- Family Medicine
- Internal Medicine
- Surgery-General
- Medical Genetics
- Neurology

Global Health Background

***In the last 5 years*, which of the following professional activities best describes your role when working in LMIC or resource-constrained settings? Select all that apply.**

- Clinical patient care
- Clinical teaching of local trainees
- Clinical teaching of trainees from HIC locations.
- Research (all types)
- Non-clinical capacity building
- Economic development
- Public health practice
- Complex humanitarian emergency response
- Quality improvement project(s)
- Clinical learning/training (for your personal professional development)
- Other

Definitions 2

For the remainder of the survey, "professional activities" refers to the activities you selected and applies to your experience in a LMIC or resource-constrained setting, unless otherwise indicated.

Emergency Interventions

Based on your experience practicing professionally in a LMIC or resource-constrained setting, what is the biggest ethical challenge for HIC professionals and trainees working in these settings?

Based on your experience practicing professionally in a LMIC or resource-constrained setting, how often do HIC professionals perform clinically beyond their scope of training when in these settings?

- Never
- Infrequently
- Sometimes
- Frequently
- Always

While practicing professionally in a LMIC or resource-constrained setting *during the last 5 years*, were you ever ASKED to perform clinical activities beyond your scope of training?

- Yes
- No

How many times *in the last 5 years* have you been ASKED to perform clinical activities beyond your scope of training?

- Once
- Twice
- Three times
- 4-10 times

More than 10 times

Too many to count

While practicing professionally in a LMIC or resource-constrained setting *during the last 5 years*, did you ever PERFORM clinical activities beyond your scope of training?

Yes

No

How many times *during the last 5 years* have you PERFORMED clinical activities beyond your scope of training?

Once

Twice

Three times

4-10 times

More than 10 times

Too many to count

Please describe briefly the most *challenging situation* in which you were asked to, or did perform, a clinical activity beyond your scope of training.

Qualitative Response

Thinking back to the *most challenging* situation in which you performed clinical activities beyond your scope of training, how did you feel at the time about the experience?

How do you feel now about the experience (described in the previous question)?

Under the same circumstances, how likely are you to perform this clinical activity (described above) again in a LMIC or resource-constrained setting?

- Not at all likely
- Slightly likely
- Moderately likely
- Very likely
- Completely likely

Why do you feel you were in a situation or situations in which you practiced clinically beyond your scope of training? Select all that apply.

- My training did not match my host's expectations.
- I was inadequately prepared to decline practicing beyond my scope of training.
- I overestimated my own capabilities.
- I had an inadequate level of supervision in-country.
- I wanted to be able to practice a procedure/technique I was not very familiar with.
- I did not seek adequate assistance when I needed it.
- Other

Overall, prior to your experiences practicing professionally in a LMIC or resource-constrained setting, were you adequately prepared on how to respond when asked to perform or when put in a position of performing clinical activities beyond your scope of training?

- No
- Yes, somewhat
- Yes, very

Global Health Skills

Do you believe it is appropriate for HIC-trained clinicians to practice beyond their scope of training when in LMIC or resource-constrained settings?

- Yes
- No
- It depends

For your previous answer, please explain why you made your selection.

Please select all procedures that you have performed in a LMIC or resource-constrained setting that are beyond your scope of training?

- Basic ultrasound
- Central venous line
- Lumbar puncture
- Paracentesis
- Thoracentesis
- Placement of intraosseous and intravenous lines
- Chest tube placement
- Anesthesia: general
- Anesthesia: local
- Endotracheal intubation
- Wound care and suturing of lacerations
- Vaginal delivery
- Complicated vaginal delivery
- Caesarian section
- Repair of perineal or vaginal lacerations
- Management of postpartum hemorrhage
- Dilation and curettage
- Basic life support obstetrics
- Neonatal resuscitation
- None

- ☐ Remediation of bag/mask ventilation
- ☐ Fracture management
- ☐ Reduction and splinting of simple dislocations
- ☐ All of the above
- ☐ Other ____

Assuming a clinician from a HIC is going to be working in a highly resource-constrained or LMIC setting without referral options, which emergency procedural skills do you believe would be most critical for that clinician to possess regardless of their specialty?

- ☐ Basic ultrasound
- ☐ Central venous line
- ☐ Lumbar puncture
- ☐ Paracentesis
- ☐ Thoracentesis
- ☐ Placement of intraosseous and intravenous lines
- ☐ Chest tube placement
- ☐ Anesthesia: general
- ☐ Anesthesia: local
- ☐ Endotracheal intubation
- ☐ Remediation of bag/mask ventilation
- ☐ Fracture management
- ☐ Reduction and splinting of simple dislocations
- ☐ Wound care and suturing of lacerations
- ☐ Vaginal delivery
- ☐ Complicated vaginal delivery
- ☐ Caesarian section
- ☐ Repair of perineal or vaginal lacerations
- ☐ Management of postpartum hemorrhage
- ☐ Management of Hypertensive Emergencies in Pregnancy
- ☐ Neonatal resuscitation
- ☐ None
- ☐ All of the above
- ☐ Other ____

Global Health Background 2

***In the last 12 months*, how much time have you spent in a LMIC or resource-constrained setting taking part in professional activities?**

	Enter a number	Select a unit of time (days/weeks/months)
	____	▼

***Over the last 5 years*, how much time on average PER YEAR have you spent in a LMIC or resource-constrained setting taking part in professional activities?**

	Enter a number	Select a unit of time (days/weeks/months)
	____	▼

LMIC Countries Select

Select all of the LMICs or resource-constrained settings where you have taken part in professional activities *in the last 5 years*. Select all that apply by holding down the CTRL button (on a PC) or the Command button (on a Mac) while making your selection.

United States of America
Canada
Afghanistan
Albania
Algeria
Andorra
Angola
Antigua and Barbuda
Argentina
Armenia

Global Health Background 3

How many times have you been to ${lm://Field/1} to participate in professional activities *during the last 5 years?*

- 1 time
- 2 times
- 3 times
- 4 times
- 5 times
- 6-10 times
- More that 10 times

Global Health Experience and Setting

Describe the type of location(s) where you have taken part in professional activities *in the last 5 years*. Select all that apply.

- Urban (city)
- Rural
- District/Village
- Peri-Urban
- Other

Describe the organization(s) with which you were most closely affiliated when taking part in professional activities *during the last 5 years*. Select all that apply.*

- Governmental
- NGOs and non-profits
- International bilateral (e.g. CDC, USAID)
- Multi-lateral (e.g. WHO, World Bank, UNICEF)
- Health care foundations (e.g. Bill & Melinda Gates Foundation, Rockefeller)
- University
- Not sure
- Other

Which of the following health care settings best describes where your professional activities have taken place *during the last 5 years.* Select all that apply.

- Community health care workers, health committees and/or volunteer community members providing basic care, follow up, screening, outreach in the home or village setting.
- Health center or dispensary staffed by non-MD (or equivalent) health workers.
- Primary care clinic staffed by doctors and/or nurses, may include basic laboratory services.
- Primary level or district hospital. Includes mainly internal medicine, obstetrics and gynecology, pediatrics, and general surgery, limited laboratory services.
- Specialty hospital or national referral center with highly specialized staff and differentiated by function. Includes psychiatry and psychology, diagnostic radiology, general and orthopedic surgery, rehabilitation, intensive care unit, specialized imaging units.intensive care unit, specialized imaging units.
- Other. Please describe.

Medical School LMIC Background

During health professional school, did you travel to a LMIC or resource-constrained setting for an elective, summer experience, or other learning/service experience?

- Yes
- No
- Not applicable

Have you returned to the LMIC or resource-constrained setting where you traveled during your training?

- No
- Once
- Twice
- Three or more times

Email

May we contact you for additional information?

- Yes
- No

Are you interested in receiving preliminary results from this survey?

- Yes
- No

Please provide your email address.

Feedback

Please include any feedback to help us improve this survey.
